# Comparison of strain imaging techniques in CRT candidates: CMR tagging, CMR feature tracking and speckle tracking echocardiography

**DOI:** 10.1007/s10554-017-1253-5

**Published:** 2017-10-17

**Authors:** Wouter M. van Everdingen, Alwin Zweerink, Robin Nijveldt, Odette A. E. Salden, Mathias Meine, Alexander H. Maass, Kevin Vernooy, Frederik J. De Lange, Albert C. van Rossum, Pierre Croisille, Patrick Clarysse, Bastiaan Geelhoed, Michiel Rienstra, Isabelle C. Van Gelder, Marc A. Vos, Cornelis P. Allaart, Maarten J. Cramer

**Affiliations:** 1 Department of Cardiology, University Medical Centre Utrecht, Utrecht, The Netherlands; 2 Department of Cardiology, and Institute for Cardiovascular Research (ICaR-VU), VU University Medical Centre, Amsterdam, The Netherlands; 3 Department of Cardiology, Thoraxcenter, University of Groningen, University Medical Centre Groningen, Groningen, The Netherlands; 4 Department of Cardiology, Maastricht University Medical Centre, Maastricht, The Netherlands; 5 Department of Cardiology, Academic Medical Centre, Amsterdam, The Netherlands; 6 Université Lyon, UJM-Saint-Etienne, INSA, CNRS UMR 5520, INSERM U1206, CREATIS, 42023 Saint-Etienne, France; 7 Department of Medical Physiology, University of Utrecht, Utrecht, The Netherlands

**Keywords:** Strain, Myocardial tagging, Feature tracking, Speckle tracking echocardiography, Dyssynchrony, Discoordination, Cardiac resynchronization therapy

## Abstract

Parameters using myocardial strain analysis may predict response to cardiac resynchronization therapy (CRT). As the agreement between currently available strain imaging modalities is unknown, three different modalities were compared. Twenty-seven CRT-candidates, prospectively included in the MARC study, underwent cardiac magnetic resonance (CMR) imaging and echocardiographic examination. Left ventricular (LV) circumferential strain was analysed with CMR tagging (CMR-TAG), CMR feature tracking (CMR-FT), and speckle tracking echocardiography (STE). Basic strain values and parameters of dyssynchrony and discoordination obtained with CMR-FT and STE were compared to CMR-TAG. Agreement of CMR-FT and CMR-TAG was overall fair, while agreement between STE and CMR-TAG was often poor. For both comparisons, agreement on discoordination parameters was highest, followed by dyssynchrony and basic strain parameters. For discoordination parameters, agreement on systolic stretch index was highest, with fair intra-class correlation coefficients (ICC) (CMR-FT: 0.58, STE: 0.55). ICC of septal systolic rebound stretch (SRS_sept_) was poor (CMR-FT: 0.41, STE: 0.30). Internal stretch factor of septal and lateral wall (ISF_sep–lat_) showed fair ICC values (CMR-FT: 0.53, STE: 0.46), while the ICC of the total LV (ISF_LV_) was fair for CMR-FT (0.55) and poor for STE (ICC: 0.32). The CURE index had a fair ICC for both comparisons (CMR-FT: 0.49, STE 0.41). Although comparison of STE to CMR-TAG was limited by methodological differences, agreement between CMR-FT and CMR-TAG was overall higher compared to STE and CMR-TAG. CMR-FT is a potential clinical alternative for CMR-TAG and STE, especially in the detection of discoordination in CRT-candidates.

**Electronic supplementary material:**

The online version of this article (doi:10.1007/s10554-017-1253-5) contains supplementary material, which is available to authorized users.

## Introduction

Cardiac resynchronization therapy (CRT) is an established treatment for patients with heart failure, reduced left ventricular (LV) ejection fraction, and a prolonged QRS caused by a left bundle branch block (LBBB) or nonspecific intraventricular conduction delay [1]. CRT aims to restore LV mechanics and improve hemodynamic by resynchronization of LV electrical activation [2]. Unfortunately, the effect of CRT is limited in 30–40% of the patients, partly due to a lack of optimal criteria for patient selection [3, 4]. In current international guidelines the selection criteria for CRT are limited to clinical parameters, ECG parameters and LV ejection fraction ≤ 35% [1]. Patient selection for CRT may be improved with additional parameters reflecting mechanical dyssynchrony or discoordination obtained with strain analysis on imaging [4–7]. These parameters reflect the LV mechanical consequences caused by an inhomogeneous electrical activation. Mechanical dyssynchrony parameters are based on timing differences between particular LV segments [8, 9]. However, these mechanical dyssynchrony parameters showed disappointing results in large multi-centre trials [9]. More promising parameters focus on discoordination, reflecting a percentage or fraction of opposing (i.e. inefficient) deformation [6, 10–12]. These parameters are determined using myocardial strain analysis, which can be obtained with several cardiac imaging techniques, including cardiac magnetic resonance imaging (CMR) with tagging (CMR-TAG), CMR cine images and a post-processing technique named feature tracking (CMR-FT), and speckle tracking echocardiography (STE) [13, 14]. Although CMR-TAG is regarded as the non-invasive ‘gold-standard’, it is generally limited to scientific applications, requiring specific imaging protocols, sequences, and dedicated post-processing software. Clinical application of CMR-FT and STE is more feasible compared to CMR-TAG, as both techniques are applicable to images obtained during standard clinical imaging protocols [14–16]. Nevertheless, both techniques (i.e. CMR-FT and STE) lack validation on strain parameters reflecting mechanical dyssynchrony and discoordination. Thus, no study has yet compared results obtained with all three techniques (i.e. CMR-TAG, CMR-FT and STE) in patients eligible for CRT. This study aims to compare circumferential strain parameters obtained with CMR-FT and STE versus gold-standard CMR-TAG in patients eligible for CRT. The comparison of indices reflecting mechanical dyssynchrony and discoordination are of specific interest.

## Materials and methods

This sub study is part of the Markers of Acute Response to CRT (MARC) study (Cohfar, CTMM, The Netherlands, clinicaltrials.gov: NCT01519908), which was designed to investigate predictors for response on CRT. The MARC study included 240 patients planned for CRT implantation in six medical centres in the Netherlands, using previously published in- and exclusion criteria [17]. Twenty-seven of the 240 patients were included in this sub-study, as these patients gave consent for an additional CMR examination including myocardial tagging in the VU university medical centre (Amsterdam, The Netherlands). All subjects gave written informed consent and the local medical ethics committees approved data collection and management. The investigation conforms to the principles outlined in the Declaration of Helsinki.

### Echocardiographic examination

Echocardiographic examinations were performed on either GE Vivid7, GE Vivid9 (General Electric Healthcare, Chicago, Illinois, USA), or Philips iE33 (Philips Medical Systems, Best, The Netherlands) ultrasound machines prior to CRT implantation by all participating centres and analysed by the echocardiographic core lab (WE and MC, UMC Utrecht, Utrecht, The Netherlands).

#### Acquisition—standard echocardiographic images

Standard echocardiographic images were obtained, including a parasternal short axis view at the papillary muscle and at the mitral valve level [18]. Image quality and frame rate (50–100 Hz) were optimized for offline speckle tracking analysis. Pulsed-wave Doppler images of the mitral valve inlet and LV outflow tract were obtained of mitral valve and aortic valve closure (AVC) to define systole.

#### Offline analysis—speckle tracking echocardiography

Echocardiographic images were exported as DICOM-files for vendor-independent strain analysis (TomTec 2D Cardiac Performance Analysis (2DCPA) version 1.2.1.2, TomTec Imaging Systems, Unterschleissheim, Germany). A region of interest was placed by user defined markers at the endocardial border. The epicardial border was excluded, as it often lacked a clear border zone. The region of interest was automatically separated into six segments. Segments were excluded if, even after repeated adjustment of the region of interest, adequate tracking was not achievable. The marker for reference length was placed at QRS onset.

STE results were exported for analysis with author written scripts for Matlab 2014b (Mathworks, Natick, MA, USA). Segmental strain curves were discarded in case of low signal-to-noise ratio as judged by two independent investigators (WE and AZ). At least two segments needed to be analysable per wall. Results of strain parameters of the septum were based on averages of maximal four septal segments (i.e. basal- and mid-level of inferoseptal and anteroseptal segments) while the lateral wall parameters were based on averages of maximal four lateral wall segments (i.e. basal- and mid-level of inferolateral and anterolateral segments). The post-processing and selection of analysable segments and averaging into one septal and one lateral wall strain curve, was similar for STE, CMR-TAG and CMR-FT.

### Cardiac magnetic resonance imaging

CMR examinations were performed on a 1.5T system (Magnetom Avanto, Siemens, Erlangen, Germany) with the use of a phased array cardiac receiver coil. Although performed on a different moment compared to STE, both standard CMR cine images for the CMR-FT analysis and CMR tagging images were obtained in the same examination.

#### Acquisition—standard CMR images

Standard CMR cine images were acquired using a retrospectively ECG-gated balanced steady-state free-precession (SSFP) sequence during end-expiratory breath holding. A stack of 8–12 consecutive short axis cine images was acquired covering the entire LV. Typical image acquisition parameters were: slice thickness 5 mm, slice gap 5 mm, echo time (TE) 1.6 ms, repetition time (TR) 3.2 ms, temporal resolution < 50 ms, in-plane spatial resolution 1.5 by 2.1 mm, flip angle 60°. The number of reconstructed temporal phases within the cardiac cycle was set at 20. Subsequently, high temporal resolution (TE 1.7 ms, TR 3.4 ms, temporal resolution ~ 15 ms) cine imaging of the LV in the three-chamber view was performed to assess the opening and closure times of the mitral and aortic valve.

#### Acquisition—CMR tagging images

Before contrast injection, tagged images were acquired at three short-axis slices (basal, mid, apical) using a complementary spatial modulation of magnetization (CSPAMM) line tagging sequence with segmented ECG-gated acquisitions and serial breath holds [16]. Typical image acquisition parameters were: slice thickness 6 mm, TE 1.7 ms, TR 3.6 ms, temporal resolution < 15 ms, in-plane spatial resolution 1.3 by 4.3 mm, flip angle 20°, tag spacing 7 mm. The number of reconstructed temporal phases within the cardiac cycle was set at 55.

#### Offline analysis—CMR tagging

Tagged CMR images were exported and analysed (AZ, RN) with the SinMod technique (inTag, v2.0, CREATIS lab, Lyon, France, run as a plug-in for OsiriX Imaging Software v6.5, Pixmeo, Switzerland) [19]. Apical slices were discarded, while basal and mid short-axis slices were used for analysis in order to match the STE slice positions. After selecting the area of interest, endocardial and epicardial contours were manually drawn in the end-systolic phase and automatically propagated. A template was placed dividing the LV in six equally sized regions, similar to STE. The myocardium was divided in three layers (i.e. endo-, mid-, epi-wall layer). Results of the mid-wall layer were used, as these results are independent of contour placement.

#### Offline analysis—CMR feature tracking

Semi-automated FT analysis software (QStrain Research Edition evaluation version 1.3.0.10, Medis, Leiden, The Netherlands) was used to analyse short-axis cine images corresponding with the mid and basal slice-location of the CMR-TAG images (AZ and RN). Apical slices were discarded to match STE. First, endo- and epicardial contours were manually drawn in both end-diastolic and end-systolic frames and propagated automatically. Both endocardial and epicardial features were included for strain analysis, resulting in myocardial strain. The LV was divided in six regions, similar to the other techniques.

#### Basic strain parameters

**Fig. 1:**
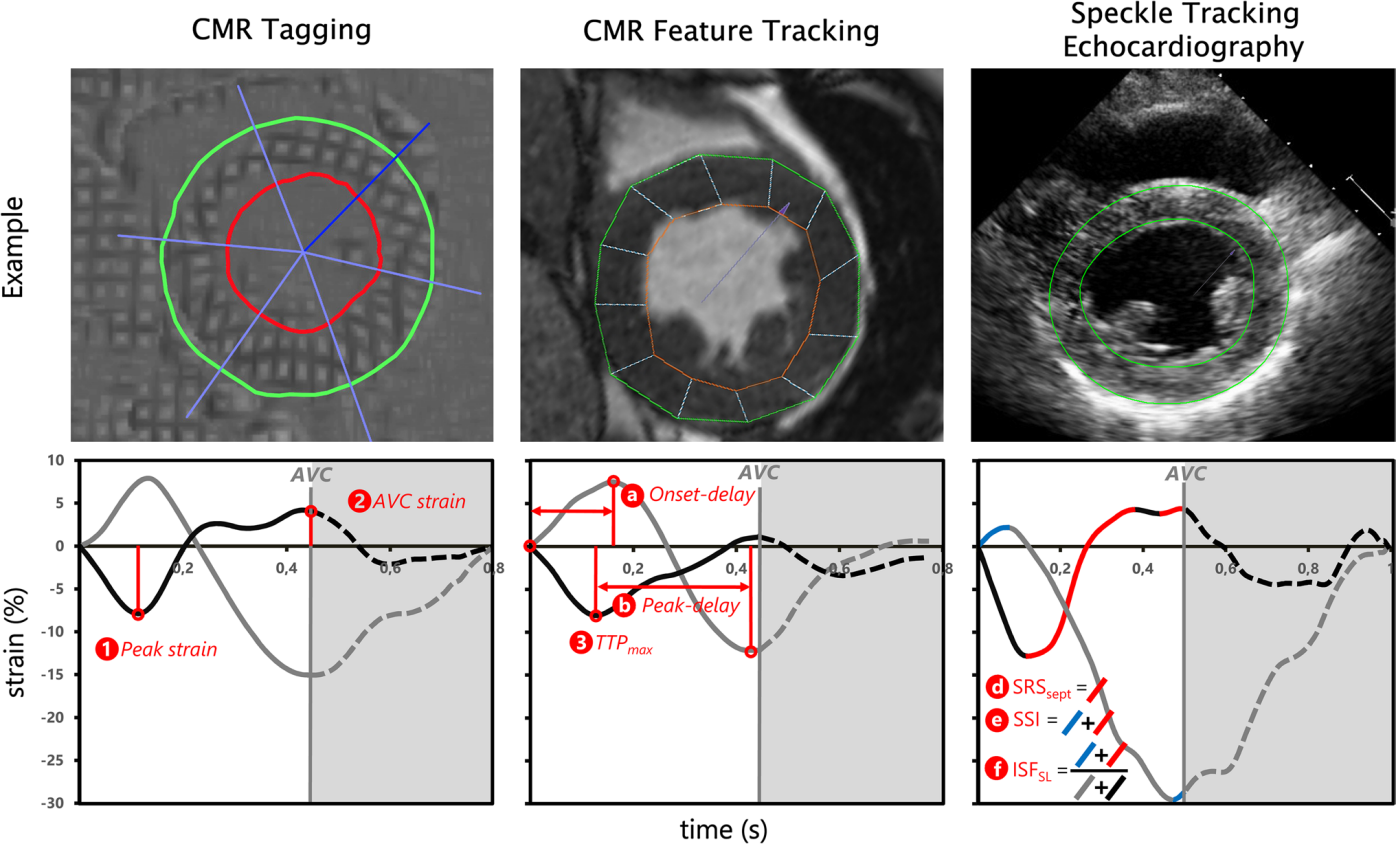
Overview of imaging techniques and corresponding myocardial strain analysis. Examples of imaging techniques (*top* row) and resulting strain signals (*bottom* row) of one specific patient. Each column represents a single technique with the corresponding strain results. Examples of derived parameters are shown per graph. Basic strain parameters are indicated with a number, dyssynchrony and discoordination parameters are indicated with a character. Strain signals of the septum (black line) and lateral wall (grey line) are given, with the aortic valve closure (grey vertical line) as end of systole. *CMR* cardiac magnetic resonance imaging, *AVC* aortic valve closure, *AVC strain* strain value at aortic valve closure, *TTP*_*max*_ time to maximal peak shortening, *onset-delay* time delay between onset of shortening of septal and lateral wall,* peak-delay* septal to lateral wall delay of TTP_max_, *SRS*_*sept*_ systolic rebound stretch of the septum, *SSI* systolic stretch index, *ISF*_*sep–lat*_ internal stretch fraction of septal and lateral wall

The following parameters were obtained for the septal and lateral wall (Fig. 1). (1) Peak strain was the maximal negative peak strain during the cardiac cycle. (2) AVC strain was defined as the strain value at aortic valve closure. (3) Time to maximal peak (TTP_max_) was the time difference between the start of the strain curve to most negative peak strain. Furthermore, (4) average systolic strain rate (i.e. average strain rate between mitral valve closure and AVC) and (5) average diastolic strain rate (i.e. average strain rate after AVC) were obtained.

#### Dyssynchrony parameters

Three parameters of dyssynchrony were analysed. (a) Onset-delay was determined as the absolute time delay between onset of shortening of the septal and lateral wall. (b) Peak delay was calculated as the absolute difference between lateral and septal wall TTP_max_. (c) The TTP_SD_ was calculated as the standard deviation of TTP_max_ of all analysable segments of the total LV.

#### Regional discoordination parameters

**Fig. 2:**
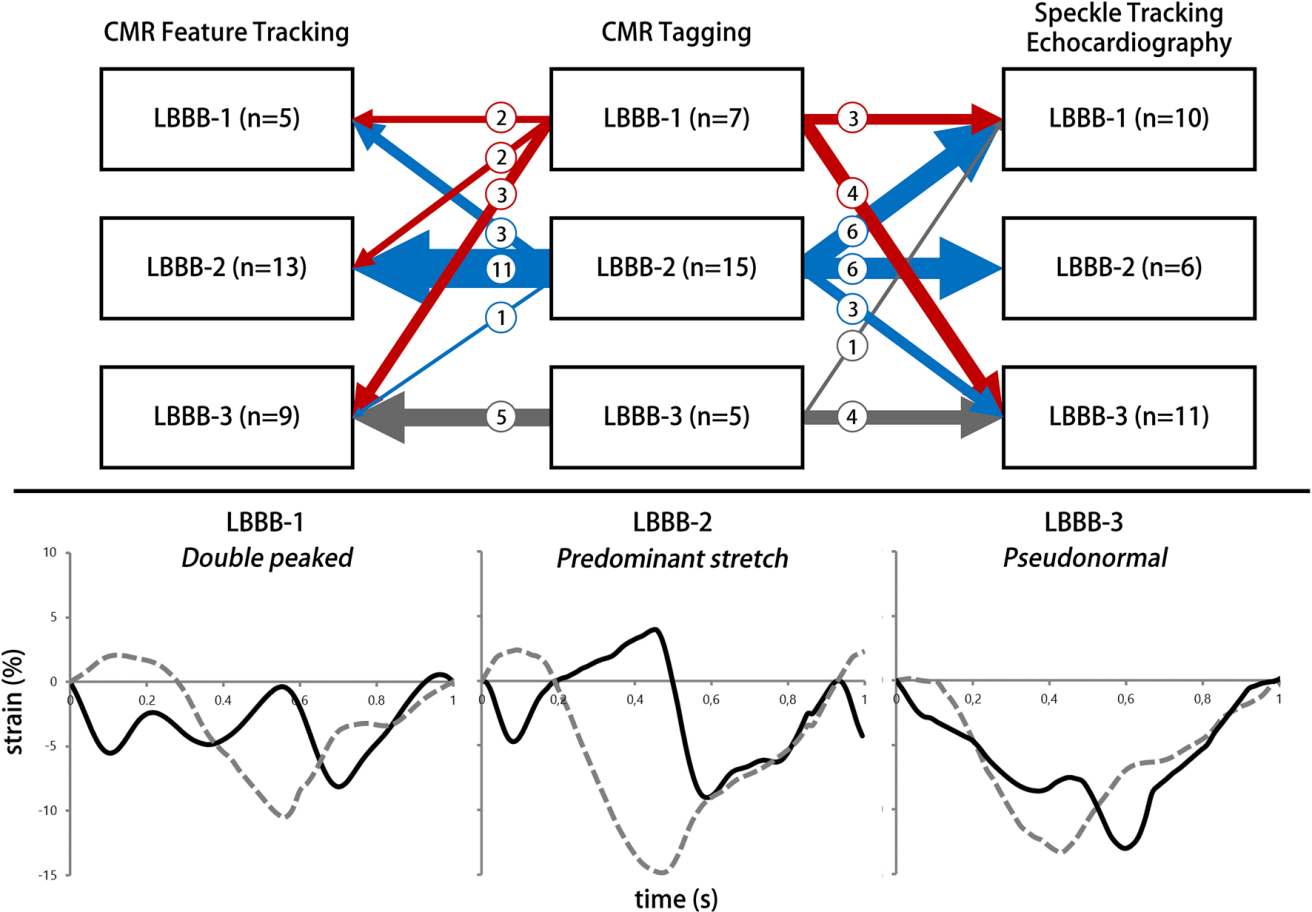
LBBB pattern categorization. Septal strain pattern categorization and distribution of strain patterns found by the three imaging techniques. The distribution per imaging technique is given vertical in the upper panel. The cross-over of patients from CMR tagging to speckle tracking echocardiography and CMR feature tracking is displayed by arrows. The thickness of the arrows matches the number of patients crossing over. The number of patients crossing over is also given by a number in each arrow. Specific examples of the three patterns are given in the lower panel. Black curve: septal strain, grey dashed curved: lateral wall strain. *CMR* cardiac magnetic resonance imaging, *LBBB-1* double peak shortening, *LBBB-2* predominant stretch, *LBBB-3* pseudo-normal shortening, *n* number of patients

Three regional discoordination parameters were analysed. (d) Systolic rebound stretch of the septum (SRS_sept_) was defined as the total amount of systolic stretch after initial shortening of the septum (Fig. 1). (e) Systolic stretch index (SSI) was calculated by adding SRS_sept_ to all systolic stretch of the lateral wall [11]. (f) Internal stretch factor (ISF) was calculated as the fraction of all systolic stretch compared to cumulative systolic shortening for the septal and lateral wall (ISF_sep–lat_). (g) Septal strain curves were categorized in three types, determined by their shape, LBBB-1: double-peaked systolic stretch, LBBB-2: early pre-ejection shortening peak followed by prominent systolic stretching and LBBB-3: pseudo normal shortening with a late-systolic shortening peak, followed by less pronounced end-systolic stretch (Fig. 2) [12].

#### Discoordination parameters of the total LV

Finally, two discoordination parameters reflecting the total LV were analysed. (h) The internal stretch factor of the total LV (ISF_LV_) was determined using all analysable segments. ISF_LV_ was determined as the total amount of stretch divided by the total amount of shortening during systole (supplemental Fig. 1) [20]. (i) Lastly, the circumferential uniformity ratio estimates (CURE) was calculated, ranging from 0 (i.e. total dyssynchrony) to 1 (i.e. perfectly synchronous) [21].

### Statistical analysis

Statistical analysis was performed (BG and MR) using R version 3.3.2 (The R foundation for Statistical Computing), and the R-packages psych version 1.5.8 (for calculation of Cohen’s kappa coefficients, ICCs and their associated p values). Results obtained with the three techniques were compared using the intra-class correlation coefficient (ICC) for absolute agreement between techniques (ICC2 according to Shrout and Fleiss) [22] and Spearman rank or Pearson correlation coefficient (R) depending on normality of data. An ICC ≥ 0.75 was classified as excellent, 0.60–0.74 as good, 0.40–0.59 as fair, and < 0.40 as poor [23]. Bland–Altman plots were made to observe the agreement between modalities. The mean difference and limits of agreement (± 1.96 standard deviation) of the Bland–Altman plot were used a reference of agreement. Lastly, Cohen’s kappa coefficient was calculated as the level of agreement between modalities on septal strain pattern categorization. A statistical result with a p value < 0.05 was deemed significant.

## Results

### Study population

Twenty-seven patients with CMR tagging images were included, of which a detailed description is given in Table 1. In these patients 94% of all segments were analysable with CMR-TAG, 87% with CMR-FT and 89% with STE. Frame rate of echocardiographic images was on average 65 ± 11 Hz, which corresponds to a temporal resolution of ~ 15 ms. Temporal resolution of CMR-TAG was ~ 14 ms, while it was ~ 40 ms for CMR-FT.

**Table 1:** Baseline characteristics

Variable	Total cohort (n = 27)
Age (years)	65.1 ± 9.7
Gender (n, male)	15 (56%)
Aetiology (n, ischemic cardiomyopathy)	7 (26%)
BMI (kg/m^2^)	26.3 ± 3.9
QRS width (ms)	183 (167–194)
Sinus rhythm (%)	100%
QRS morphology (n)	
LBBB	21 (81%)
IVCD	6 (19%)
NYHA class (n)	
II	17 (63%)
III	10 (37%)
Medication (n)	
Beta-blockers	23 (85%)
Diuretics	22 (81%)
ACE/ATII inhibitors	17 (63%)
Aldosterone antagonists	10 (37%)
CMR—LVEDV (ml)	317 ± 100
CMR—LVESV (ml)	239 ± 99
CMR—LVEF (%)	26.7 ± 8.8
CMR—LV mass (gr)	131 (118–157)

Mean and standard deviation are given with ± symbol, median and interquartile range between brackets

*BMI* body surface mass index, *CMR* cardiac magnetic resonance imaging, *LBBB* left bundle branch block, *IVCD* intraventricular conduction delay, *NYHA* New York Heart Association, *ATII* angiotensin receptor II, *LVEDV* left ventricular end-diastolic volume, *LVESV* left ventricular end-systolic volume, *LVEF* left ventricular ejection fraction, *LV* left ventricular

### Basic strain parameters

**Fig. 3:**
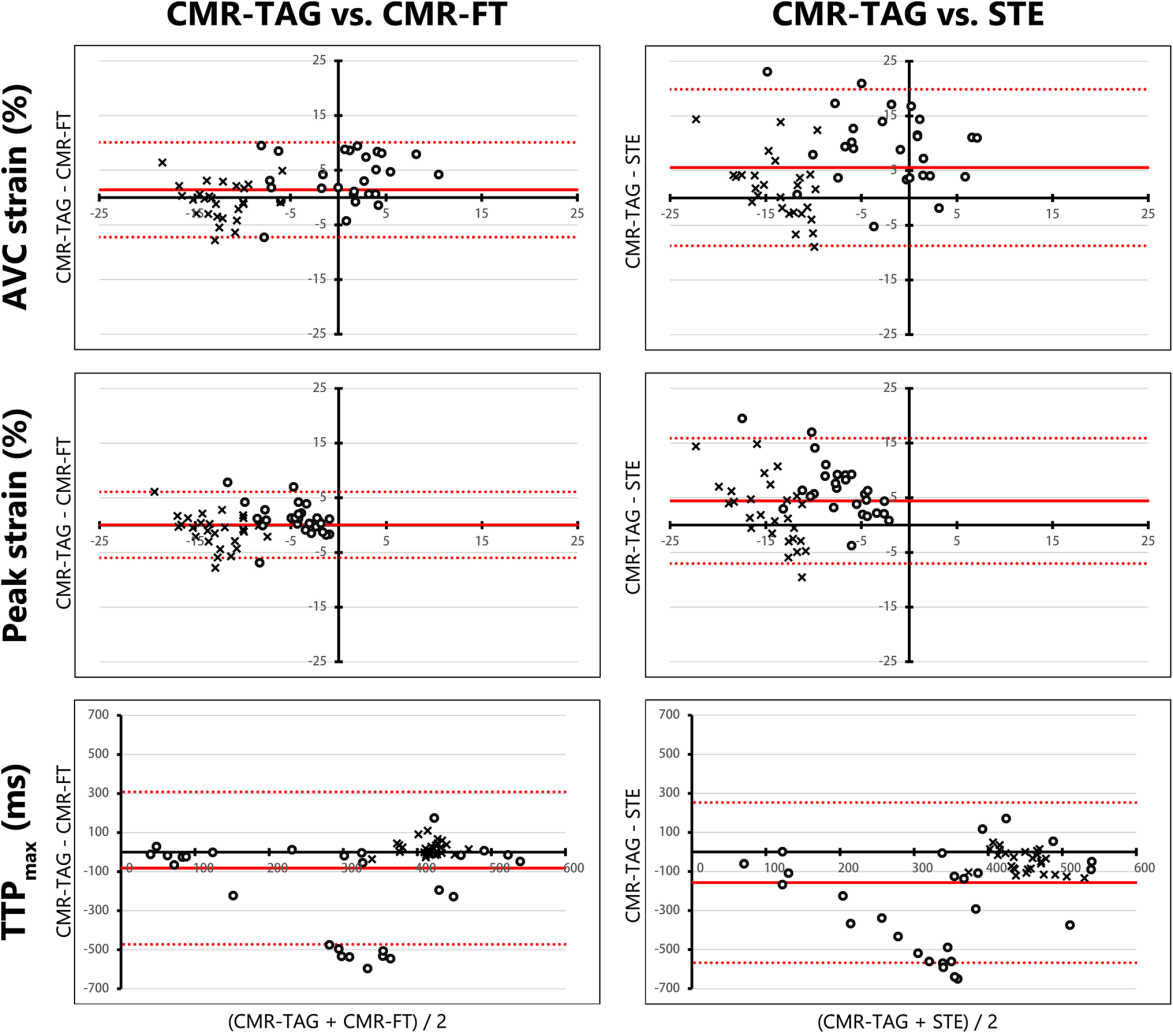
Bland–Altman plots of basic strain parameters. Bland–Altman plots for CMR-TAG versus CMR-FT and CMR-TAG versus STE of three basic strain parameters. The mean of two techniques is plotted on the x-axis and the difference on the y-axis. The mean difference is displayed as a solid red line, while the limits of agreement are displayed as dotted red lines. Septal values are given as dots, while lateral wall values are given as crosses. *AVC strain* strain at aortic valve closure time, *Peak strain* highest negative peak strain value, *TTP*_*max*_ time to maximal peak strain, *CMR* cardiac magnetic resonance imaging, *TAG* tagging, *FT* feature tracking, *STE* speckle tracking echocardiography

Overall, agreement of CMR-TAG and CMR-FT was higher compared to agreement of CMR-TAG and STE for basic strain parameters. This applied for ICC values, Bland–Altman characteristics and the correlation coefficient (R) (Table 3). (1) For CMR-FT AVC strain of the septum was fair (ICC 0.55, R 0.67), while it was poor for STE (ICC 0.23, R 0.47). The ICC of AVC strain of the lateral wall was fair for CMR-FT (ICC 0.50, R 0.50) and poor for STE (ICC 0.08, R 0.10). (2) Peak strain of the septum had a fair ICC for CMR-FT (ICC 0.58, R 0.55) and a poor ICC for STE (ICC 0.155, 0.42). Lateral wall peak strain also had a fair ICC for CMR-FT (ICC 0.54, R 0.59) and a poor ICC for STE (ICC 0.01, R 0.02). (3) TTP_max_ of the septal and lateral wall showed an apparent wide distribution in the Bland–Altman plots for both comparisons (Fig. 3). Septal TTP_max_ caused a large spread in results, while the lateral wall TTP_max_ were more similar for both comparisons. Although ICC for TTP_max_ was poor for all comparisons, CMR-FT showed better agreement with CMR-TAG compared to STE for the septum (CMR-FT: ICC 0.17, R 0.11 and STE: ICC 0.00, R −0.16) and the lateral wall (CMR-FT: ICC 0.34, R 0.40 and STE: ICC 0.13, R 0.23). (4) Systolic strain rate showed comparable results to AVC strain. For CMR-FT systolic strain rate of the septum was fair (ICC 0.56, R 0.66), while it was poor for STE (ICC 0.25, R 0.45). ICC of the systolic strain rate of the lateral wall was fair for CMR-FT (ICC 0.575, R 0.58) and poor for STE (ICC 0.05, R 0.05). (5) Diastolic strain rate showed good ICC for the septal (ICC 0.64, R 0.66) and excellent ICC for the lateral wall (ICC 0.82, 0.82) for CMR-TAG versus CMR-FT. ICC of diastolic strain rate was poor for both walls comparing CMR-TAG and STE (septum: ICC 0.34, R 0.50, lateral wall: ICC 0.23, R 0.38).

**Table 2:** Strain parameters of each myocardial strain analysis modality

	CMR tagging	CMR feature tracking	STE
Basic strain septum			
(1) AVC strain septum (%)	2.4 ± 5.8	− 1.1 ± 5.0	− 6.8 ± 7.1
(2) Peak strain septum (%)	− 4.0 ± 2.8	− 5.1 ± 3.6	− 10.4 ± 5.4
(3) TTP_max_ septum (ms)	195 ± 179	379 ± 211	459 ± 173
(4) Systolic strain rate septum (%/s)	5.4 ± 16.4	− 3.0 ± 12.9	− 15.9 ± 16.1
(5) Diastolic strain rate septum (%/s)	− 1.1 ± 10.8	2.1 ± 10.9	− 1.9 ± 29.0
Basic strain lateral wall			
(1) AVC strain lateral (%)	− 12.6 ± 3.2	− 12.0 ± 3.5	− 14.5 ± 5.3
(2) Peak strain lateral (%)	− 13.4 ± 2.7	− 12.4 ± 3.6	− 15.8 ± 5.5
(3) TTP_max_ lateral (ms)	424 ± 33	404 ± 31	474 ± 52
(4) Systolic strain rate lateral (%/s)	− 32.2 ± 7.9	− 30.8 ± 9.3	− 32.8 ± 11.9
(5) Diastolic strain rate lateral (%/s)	29.7 ± 13.2	27.8 ± 13.7	12.5 ± 25.0
Dyssynchrony			
(a) Onset-delay (ms)	55 ± 25	58 ± 46	57 ± 61
(b) Peak-delay (ms)	268 ± 127	189 ± 104	144 ± 104
(c) TTP_SD_ (ms)	149 ± 48	159 ± 44	149 ± 52
Discoordination septal and lateral wall			
(d) SRS_sept_ (%)	7.2 ± 4.5	3.8 ± 2.6	3.6 ± 3.9
(e) SSI (%)	8.7 ± 5.5	5.1 ± 3.8	5.0 ± 4.4
(f) ISF_sep–lat_	0.43 ± 0.25	0.29 ± 0.21	0.25 ± 0.16
(g) Septal strain patterns (n, %)			
LBBB-1	7 (26)	5 (19)	10 (37)
LBBB-2	15 (56)	13 (48)	6 (22)
LBBB-3	5 (19)	9 (33)	11 (41)
Discoordination total LV			
(h) ISF_LV_	0.46 ± 0.22	0.35 ± 0.17	0.37 ± 0.13
(i) CURE	0.81 ± 0.09	0.77 ± 0.09	0.78 ± 0.06

*CMR* cardiac magnetic resonance imaging, *TAG* tagging, *FT* feature tracking, *STE* speckle tracking echocardiography, *AVC strain* strain value at aortic valve closure, *TTP*_*max*_ time to maximal peak shortening, *onset-delay* time delay between onset of shortening of septal and lateral wall, *peak-delay* septal to lateral wall delay of TTP_max_, *TTP*_*SD*_ standard deviation if time to peak max of all segments, *SRS*_*sept*_ septal systolic rebound stretch, *SSI* systolic stretch index, *ISF*_*sep–lat*_ internal stretch factor of septum and lateral wall, *ISF*_*LV*_ internal stretch factor of all left ventricular segments, *CURE* circumferential uniformity ratio estimates

### Dyssynchrony parameters

(a) Onset delay was quite similar for CMR-TAG and CMR-FT, with a mean difference in the Bland–Altman plot of −2.5 ms (Supplemental Fig. 2). The corresponding ICC was fair (ICC 0.42, R 0.23). CMR-TAG versus STE also had a low mean difference of -1.9 ms, although the limits of agreement where larger, combined with a poor ICC (ICC 0.024, R −0.08). (b) Peak delay of CMR-TAG was overall larger compared to CMR-FT and STE (Supplemental Fig. 2). ICC was fair for CMR-FT (ICC 0.45, R 0.46), and poor for STE (ICC 0.23, R 0.27). TTP_SD_ (c) showed a fair ICC for CMR-FT (ICC 0.46, R 0.49), and poor ICC for STE (ICC 0.20, R 0.19).

### Regional discoordination parameters

**Fig. 4:**
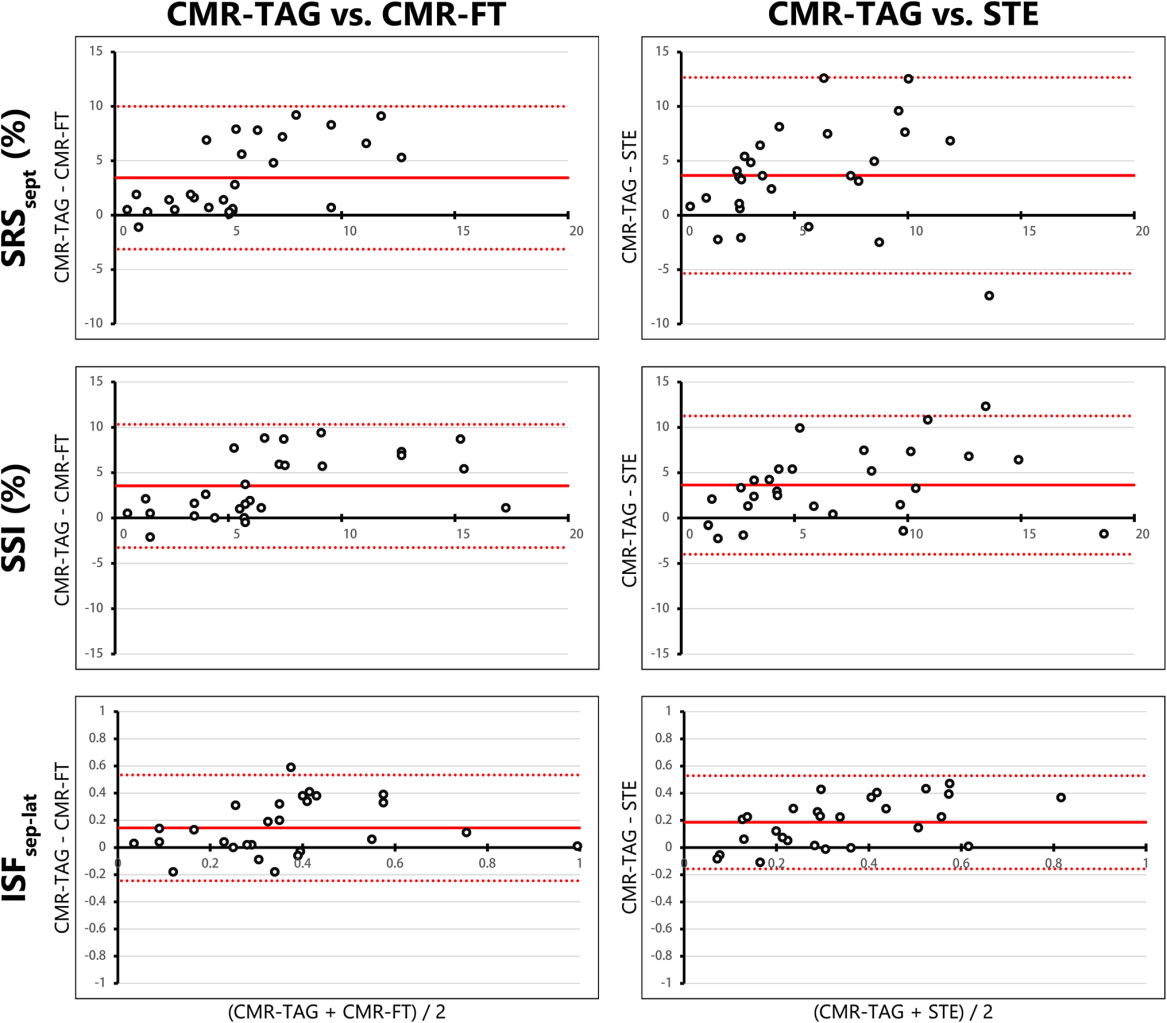
Bland–Altman plots of regional discoordination parameters. Bland–Altman plots for CMR-TAG versus CMR-FT and CMR-TAG versus STE of regional discoordination parameters (i.e. SRS_sept_, SSI and ISF_sep–lat_). The mean value of one patient analysed with the two techniques is plotted on the x-axis and the difference on the y-axis. The mean difference is displayed as a solid red line, while the limits of agreement are displayed as dotted red lines. *SRS*_*sept*_ septal systolic rebound stretch, *SSI* systolic stretch index, *ISF*_*sep–lat*_ internal stretch factor of septum and lateral wall, *CMR* cardiac magnetic resonance imaging, *TAG* tagging, *FT* feature tracking, *STE* speckle tracking echocardiography

(d) SRS_sept_ showed a fair ICC for CMR-FT (ICC 0.41, R 0.65), while agreement was poor for STE (ICC 0.30, R 0.41). CMR-TAG showed overall higher values for SRS_sept_ compared to both other imaging techniques. The difference of CMR-TAG to CMR-FT and STE were mostly positive, indicating an underestimation by CMR-FT and STE (Fig. 4). (e) SSI also showed an overall underestimation by CMR-FT and STE compared to CMR-TAG. Agreement on SSI was fair for CMR-FT (ICC 0.58, R 0.68) and STE (ICC 0.55, R 0.70). (f) ISF_sep–lat_ was comparable between techniques, ICC’s of both CMR-FT (ICC 0.53, R 0.45) and STE (ICC 0.46, R 0.69) were fair. Overall values were still lower by CMR-FT and STE compared to CMR-TAG (Fig. 4). For septal strain patterns (g) the kappa value of CMR-TAG versus CMR-FT (0.465 p < 0.001) was higher compared to the kappa of CMR-TAG versus STE (0.265, p < 0.001). The number of patients crossing over from LBBB-1 or LBBB-2 on the one hand, and LBBB-3 on the other, using CMR-TAG and CMR-FT is rather low (n = 4, 15%), especially compared to CMR-TAG and STE (n = 8, 30%) (Fig. 2).

### Discoordination parameters of the total LV

**Fig. 5:**
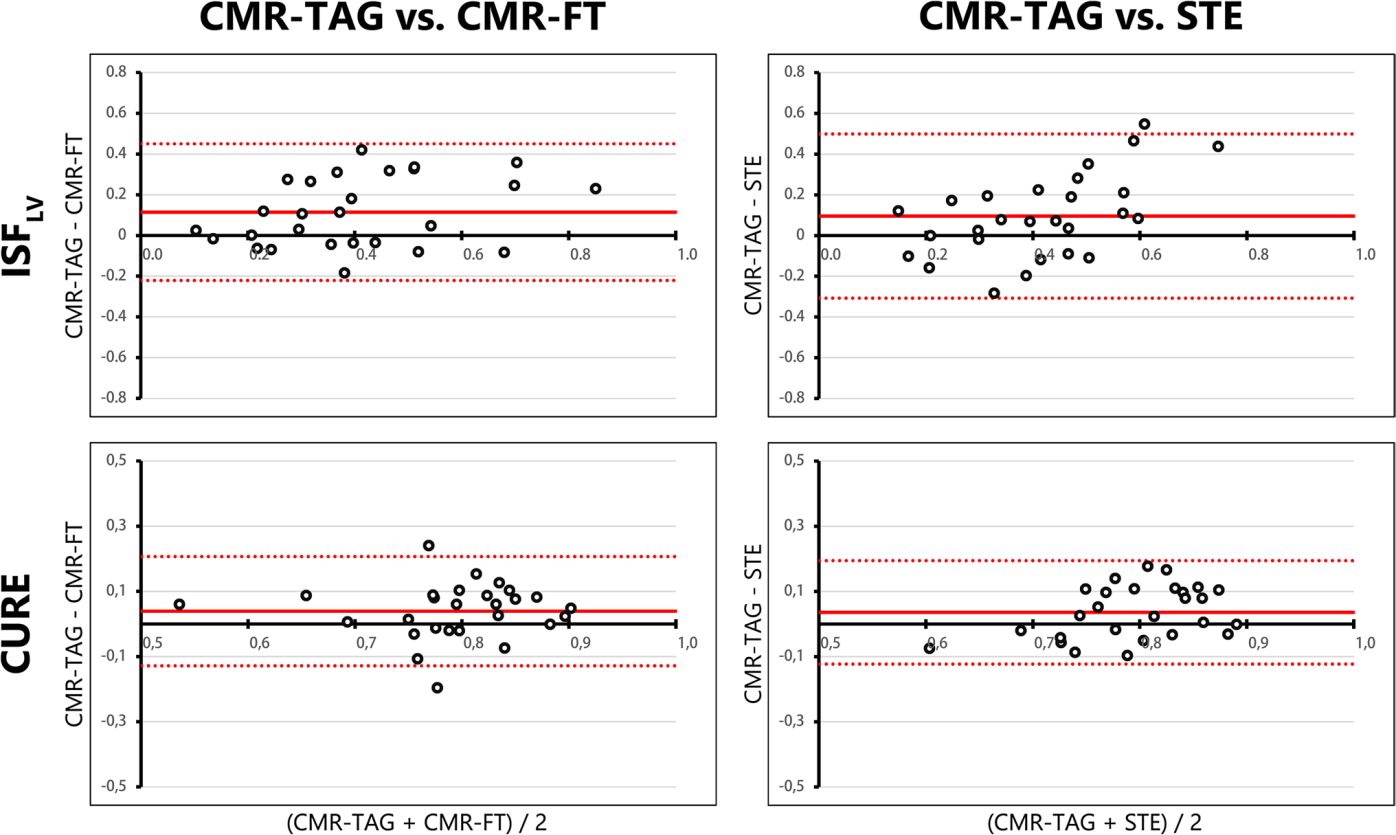
Bland–Altman plots of discoordination parameters of the total LV. Bland–Altman plots for CMR-TAG versus CMR-FT and CMR-TAG versus STE of two discoordination parameters, obtained from the total LV. The mean value of one patient analysed with the two techniques is plotted on the x-axis and the difference on the y-axis. The mean difference is displayed as a solid red line, while the limits of agreement are displayed as dotted red lines. *ISF*_*LV*_ internal stretch factor of the total LV, *CURE* circumferential uniformity ratio estimates, *LV* left ventricle, *CMR* cardiac magnetic resonance imaging, *TAG* tagging, *FT* feature tracking, *STE* speckle tracking echocardiography

ICC of ISF_LV_ (h) of CMR-FT (ICC 0.55, R 0.66) was the highest off all dyssynchrony and discoordination parameters. Both ICC and R values were lower for STE (ICC 0.32, R 0.42). The CURE index (i) showed rather comparable values between techniques (Fig. 5) with relative narrow limits of agreement in the Bland–Altman plot. Both CMR-FT (ICC 0.485, R 0.37) and STE (ICC 0.41, R 0.36) resulted in a fair ICC value for CURE compared to CMR-TAG (Table 3).

**Table 3:** Intra-class correlation and correlation of CMR tagging versus CMR feature tracking and CMR tagging versus speckle tracking echocardiography

	CMR-TAG vs. CMR-FT (n = 27)		CMR-TAG vs. STE (n = 27)	
	ICC (95% CI)	R	ICC (95% CI)	R
Basic strain septum				
(1) AVC strain septum (%)	0.55 (0.09–0.79)	0.67^‡^	0.23 (− 0.10–0.56)	0.47*
(2) Peak strain septum (%)	0.58 (0.26–0.78)	0.55^†^	0.155 (− 0.10–0.45)	0.42*
(3) TTP_max_ septum (ms)	0.17 (− 0.11–0.47)	0.11	0.00 (− 0.15–0.22)	− 0.16
(4) Systolic strain rate septum (%/s)	0.56 (0.16–0.79)	0.66^‡^	0.25 (− 0.10–0.57)	0.45*
(5) Diastolic strain rate septum (%/s)	0.64 (0.35–0.82)	0.66^‡^	0.34 (− 0.05–0.635)	0.50^†^
Basic strain lateral wall				
(1) AVC strain lateral (%)	0.50 (0.16–0.74)	0.50^†^	0.08 (− 0.27–0.43)	0.10
(2) Peak strain lateral (%)	0.54 (0.22–0.76)	0.59^†^	0.01 (− 0.31–0.36)	0.02
(3) TTP_max_ lateral (ms)	0.34 (0.00–0.63)	0.40*	0.13 (− 0.12–0.41)	0.23
(4) Systolic strain rate lateral (%/s)	0.575 (0.26–0.78)	0.58^†^	0.05 (− 0.35–0.42)	0.05
(5) Diastolic strain rate lateral (%/s)	0.82 (0.65–0.91)	0.82^‡^	0.23 (− 0.08–0.53)	0.38
Dyssynchrony				
(a) Onset-delay (ms)	0.42 (0.05–0.69)	0.23	0.024 (− 0.37–0.40)	− 0.08
(b) Peak-delay (ms)	0.45 (0.045–0.715)	0.46*	0.23 (− 0.09–0.53)	0.27
(c) TTP_SD_ (ms)	0.46 (0.11–0.71)	0.49^†^	0.20 (− 0.20–0.54)	0.19
Regional discoordination				
(d) SRS_sept_ (%)	0.41 (− 0.06–0.72)	0.65^‡^	0.30 (− 0.05–0.60)	0.41*
(e) SSI (%)	0.58 (0.00–0.83)	0.68^‡^	0.55 (0.02–0.81)	0.70^‡^
(f) ISF_sep–lat_	0.53 (0.10–0.77)	0.45*	0.46 (− 0.06–0.76)	0.69^‡^
Discoordination total LV				
(h) ISF_LV_	0.55 (0.15–0.78)	0.66^‡^	0.32 (− 0.02–0.61)	0.42*
(i) CURE	0.485 (0.145–0.725)	0.37	0.41 (0.06–0.67)	0.36

*CI* confidence interval, *ICC* intra-class correlation coefficient, *R* correlation coefficient, for other abbreviations see Table 2

P values for statistical significance of R-values are given with: *p value < 0.05, ^†^p value < 0.01, ^‡^p value < 0.001

## Discussion

This study explores the comparison of strain parameters in CRT candidates of two widely available strain analysis techniques, speckle tracking echocardiography and CMR feature tracking, with gold-standard CMR tagging. While most basic strain and dyssynchrony parameters differed substantially between techniques, there were apparent similarities found for discoordination parameters. This finding is promising, as discoordination parameters are potential predictors for CRT response [10, 11, 20]. The CMR-based techniques (i.e. CMR-TAG and CMR-FT) showed the highest agreement, shown in fair ICC values, higher R values, and relative narrow limits of agreement of the Bland–Altman plots. STE mostly had a poor agreement with CMR-TAG. CMR-FT may therefore be a valuable alternative to CMR-TAG for analysis of discoordination parameters in patients eligible for CRT.

### Comparison of imaging techniques

To the best of our knowledge, this is the first study to compare strain parameters between different strain analysis techniques in a CRT patient population. The overall agreement between CMR-FT and CMR-TAG was higher compared to the agreement between STE and CMR-TAG. We would like to discuss three considerations to ascribe this difference. Firstly, STE uses a different imaging source, while both CMR-FT and CMR-TAG are obtained with the same imaging modality. Second, as part of the protocol, echocardiographic examinations and CMR scans were not performed on the same day. Therefore, physiological differences, such as loading conditions and heart rate, may have interfered with agreement of STE and CMR-TAG. Third, the imaging plane used for CMR and echocardiography is possibly different. Echocardiographic parasternal short-axis views were obtained from a single intercostal position, angulating the echo probe to the mitral valve annulus plane and the papillary muscle plane. These imaging planes may thereby be partly oblique, while CMR imaging planes were ‘true’ short-axis views. Furthermore, CMR-FT and CMR-TAG images were acquired on the almost exact same slice position, while the anatomical plane of STE images may be different. Another factor causing discrepancies between techniques is the specific manufacturer used for strain analysis with either CMR-TAG, CMR-FT, or STE [24]. Results of CMR-TAG, CMR-FT, and STE are contemporary, as they are dependent on specific analysis algorithms which are constantly under development. Although earlier studies show less favourable agreement of CMR-TAG and CMR-FT,[25, 26] recent developments are more promising [27, 28]. This trend is in accordance with our results, as we found that CMR-FT had fair agreement with CMR-TAG. However, further improvements are necessary, as results obtained with different imaging techniques can still differ largely for the individual patient. These differences may have underestimated the agreement between STE and CMR-TAG, compared to CMR-TAG versus CMR-FT. Nevertheless, echocardiography has its known limitations. High quality images are required for reliable strain analysis with STE,[29, 30] but can be difficult in this selection of patients. Frame rate is directly related to the temporal resolution, which is often high in echocardiographic images, especially compared to the relative low frame rate of standard cine images used for CMR-FT. A low frame rate causes under sampling and may lead to misinterpretation of peak and time-to-peak values in strain signals [29]. The frame rate of CMR-TAG was relatively high and comparable to STE in our study. Therefore, CMR-TAG may be considered a true gold-standard technique in this study, as imaging quality and frame rate of the implemented tagging protocol were optimized.

### Assessment of strain parameters

Peak strain parameters showed fair correlation, especially between CMR techniques, except for timing indices of the septum. The maximal peak of septal strain can shift easily in case of dyssynchrony, as there are often multiple peaks (e.g. LBBB-1 and LBBB-2 patterns). Changes in absolute strain values of these peaks can drastically change TTP_max_. In previous studies, most dyssynchrony and discoordination parameters have been primarily analysed with a single imaging technique. While some (i.e. CURE and ISF_LV_) are predominantly used in CMR-based studies,[20] others (i.e. SRS_sept_, peak-delay and septal strain patterns) are primarily derived with STE [12]. In our study, basic strain parameters, and more complex parameters of mechanical dyssynchrony showed apparent variations among the three techniques. However, the three techniques did show fair agreement on discoordination parameters. This indicates that these parameters adequately reflect mechanical discoordination and that they are detectable by multiple modalities. Discoordination parameters are promising as predictors for CRT response [10, 11, 20]. The predictive value of discoordination parameters is even known in combination with electrocardiographic parameters [6, 11]. ISF_LV_ and CURE are predictors of CRT response and use information of all available LV segments,[31] therefore reflecting total LV discoordination [20, 21]. These parameters are also less susceptible to outliers compared to basic strain parameters, as they contain information on all segments [20, 21]. Parameters being calculated using averages of multiple segments (i.e. SRS_sept_, SSI) also showed fair agreement between modalities. Obtaining deformation characteristics using averages of multiple segments may therefore reduce noise and measurement variability. Specific pre-specified septal strain patterns are known to predict CRT response, as LBBB-1 and LBBB-2 patterns are associated with volumetric response after CRT, while LBBB-3 is not [12, 32]. The relative high agreement between CMR-TAG and CMR-FT on LBBB-1 and LBBB-2 on the one hand, and LBBB-3 on the other is therefore promising for further implementation of septal strain pattern categorization using CMR.

### Myocardial strain orientation

STE parameters are mainly validated with longitudinal strain,[33, 34] while CMR is predominantly based on circumferential strain [20, 21]. Circumferential strain is more intuitive, as mid-myocardial fibres are orientated in the circumferential direction and short-axis images represent all segments distributed around the LV at each level (i.e. basal, mid or apical) [35]. The method of determining circumferential strain calculation differs between the three methods. Both the CMR-FT and STE software track specific myocardial details, respectively ‘features’ and ‘speckles’, of the endo- and epicardial border [14]. The specific wall layer used for strain analysis differed between techniques. The results of the endocardial layer were used for STE, as the epicardial layer often lacked an appropriate border zone. Strain values of CMR-FT were a product of endocardial and epicardial strain. This is in contrast to CMR-TAG, of which strain of the mid-wall layer was used [19]. The difference between the approaches may have biased the overall level of agreement. Endocardial strain is known to give higher peak values compared to epicardial strain [36], and might also be higher than midmyocardial values, which can be appreciated in the positive mean difference between CMR-TAG and STE on peak strain and AVC-strain in the Bland–Altman results. This difference may have also affected the agreement of dyssynchrony and discoordination parameters.

### Limitations

CMR imaging with myocardial tagging was performed in a small subset of patients from the MARC study, which may have given outliers a relatively large effect on results. The patient population was moreover limited to patients eligible for CRT, reducing variability in measurements. These results should therefore be validated in a larger cohort. However, strain measurements are of particular interest in this specific population to improve patient selection for CRT. As mentioned, the study protocol has also influenced results, as echocardiographic and CMR examination were not performed on the same day. Moreover, differences in imaging plane between CMR and STE are possible and strain analysis was not performed on the same wall layers. ECG triggering differs between imaging techniques, as ECG electrodes were repositioned between examinations and a different lead may have been used. Moreover, ECG triggering of STE was placed at QRS onset, while the top of the R wave is used for CMR. ECG triggering affects the reference value and may have affected subsequent values of timing and absolute changes. While STE relied on end-diastolic region of interest placement, CMR-FT used both end-systolic and end-diastolic region of interests to determine myocardial strain. The reliability of CMR-FT may therefore be higher. Echocardiography was moreover obtained with ultrasound machines from two vendors, possibly introducing differences in source data. The overall lower agreement of CMR-TAG and STE should therefore be appreciated carefully. STE was performed with circumferential strain obtained from short axis images, for a more direct comparison between techniques. While circumferential strain is widely used in scientific publications, standardization of algorithms of STE has also mainly been done for longitudinal strain [5, 34]. Longitudinal strain assessed with STE may therefore have a higher reliability and reproducibility compared to circumferential strain. The effect of using longitudinal or circumferential strain derived with STE for prediction of CRT response deserves attention in future work.

### Clinical application

The overall reasonable agreement between CMR-TAG and CMR-FT is promising for clinical application. CMR-FT might be a reasonable alternative for CMR-TAG and STE, as suitable CMR cine images are more easily available in clinical practice, compared to the highly specialized CMR-TAG protocols. Detection of mechanical discoordination with CMR-FT is a valuable addition to CMR, which already constitutes an important imaging tool in CRT-candidates for accurate determination of the LV ejection fraction and scar tissue localization [31]. On the other hand, a portable and bed-side tool like STE might have the highest clinical applicability, of which most discoordination parameters also showed fair agreement compared to the CMR-TAG. The reasonable agreement of the three techniques on mechanical discoordination parameters is moreover promising for the prediction of response to CRT. The implemented discoordination parameters were previously associated with CRT response in single centre studies [10, 11, 20]. However, previous markers of CRT response failed to take the final step to clinical application, partly because validation to gold-standard techniques was missing [9]. As the specific methods and modality may slightly differ from previous publications, further studies are needed for implementation into clinical practice. Future studies will focus on the predictive value of these parameters using follow-up data in this specific population.

## Conclusions

In conclusion, comparison of strain analysis techniques showed that CMR-FT had an overall fair agreement with gold-standard CMR-TAG. Although agreement between STE and CMR-TAG was overall lower, direct comparison was limited by technical and methodological differences. The agreement was highest for parameters of mechanical discoordination, compared to basic strain or dyssynchrony parameters. CMR-FT is therefore a potentially valuable clinical alternative for CMR-TAG and STE, especially in the evaluation of mechanical discoordination in CRT-candidates.

## Electronic supplementary material

Below is the link to the electronic supplementary material.

Supplementary material 1 (TIF 4134 KB)

Supplementary material 2 (TIF 1316 KB)

Supplementary material 3 (DOCX 17 KB)

## Abbreviations

AVC strain: Strain value at aortic valve closure
AVC: Aortic valve closure
CMR: Cardiac magnetic resonance imaging
CMR-FT: Cardiac magnetic resonance feature tracking
CMR-TAG: Cardiac magnetic resonance myocardial tagging
CRT: Cardiac resynchronization therapy
CSPAMM: Complementary spatial modulation of magnetization
CURE: Circumferential uniformity ratio estimates.
ICC: Intra-class correlation coefficient
ISF_LV_: Internal stretch factor of all left ventricular segments
ISF_sep–lat_: Internal stretch factor of septum and lateral wall
LBBB: Left bundle branch block
LV: Left ventricle
LVEDV: Left ventricular end-diastolic volume
LVEF: Left ventricular ejection fraction
LVESV: Left ventricular end-systolic volume
MARC: Markers of response to cardiac resynchronization therapy
NYHA: New York Heart Association
Onset-delay: Time delay between onset of shortening of septal and lateral wall
Peak-delay: Septal to lateral wall delay of time to maximal peak shortening
R: Correlation coefficient
RVEF: Right ventricular ejection fraction.
SRS_sept_: Septal systolic rebound stretch
SSFP: Steady-state free-precession
SSI: Systolic stretch index
STE: Speckle tracking echocardiography
TE: Echo time
TR: Repetition time
TTP_max_: Time to maximal peak shortening
TTP_SD_: Standard deviation if time to peak max of all segments
