# Supplementary figures and images for: Comparison of strain imaging techniques in CRT candidates: CMR tagging, CMR feature tracking and speckle tracking echocardiography
Source: Int J Cardiovasc Imaging. 2017 Oct 17;34(3):443–56. doi: 10.1007/s10554-017-1253-5 (PMC5847211; doi:10.1007/s10554-017-1253-5)

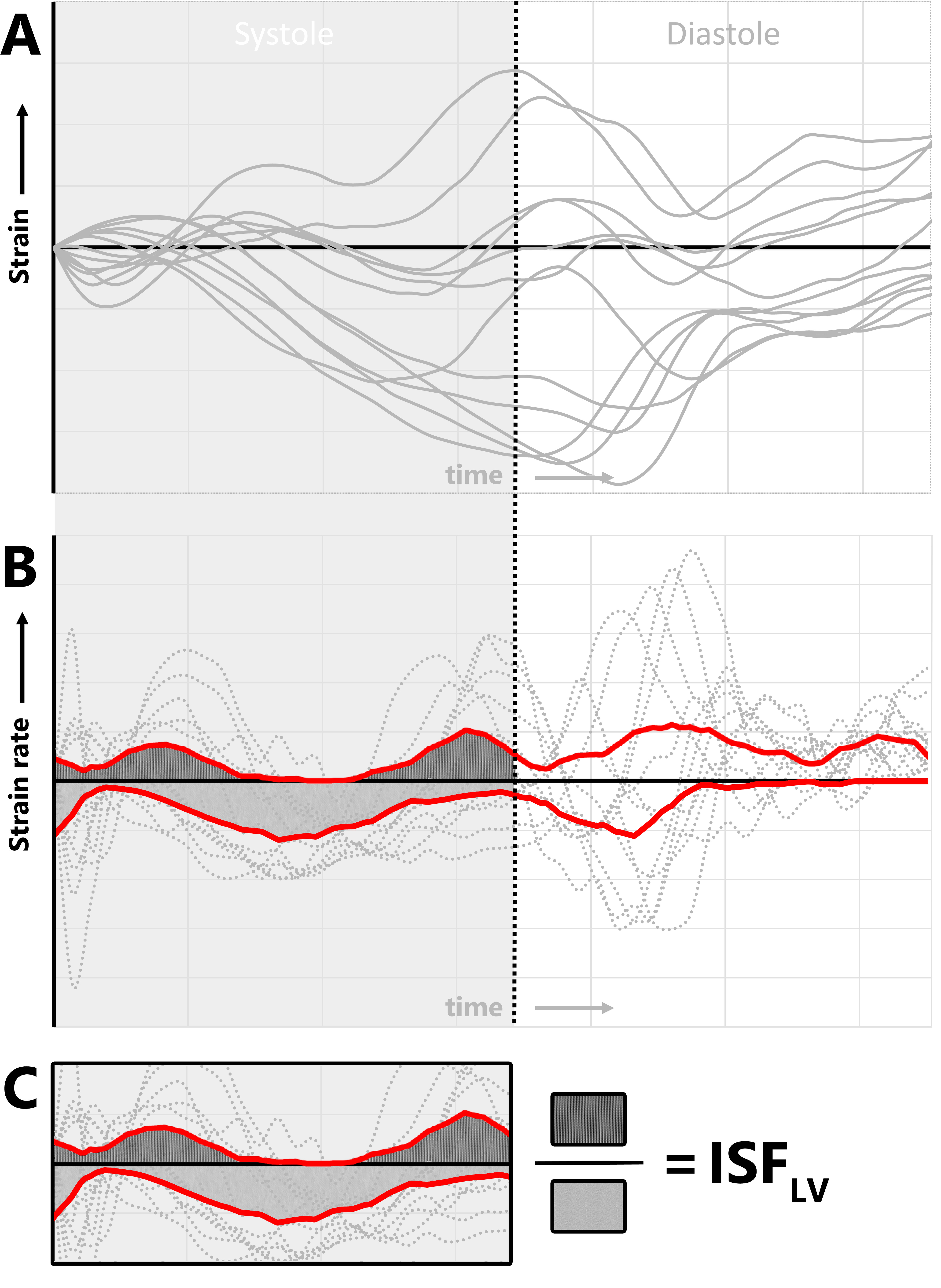

Supplement: Supplementary file 1 — Supplementary material 1 (TIF 4134 KB) [file 10554_2017_1253_MOESM1_ESM.tif]

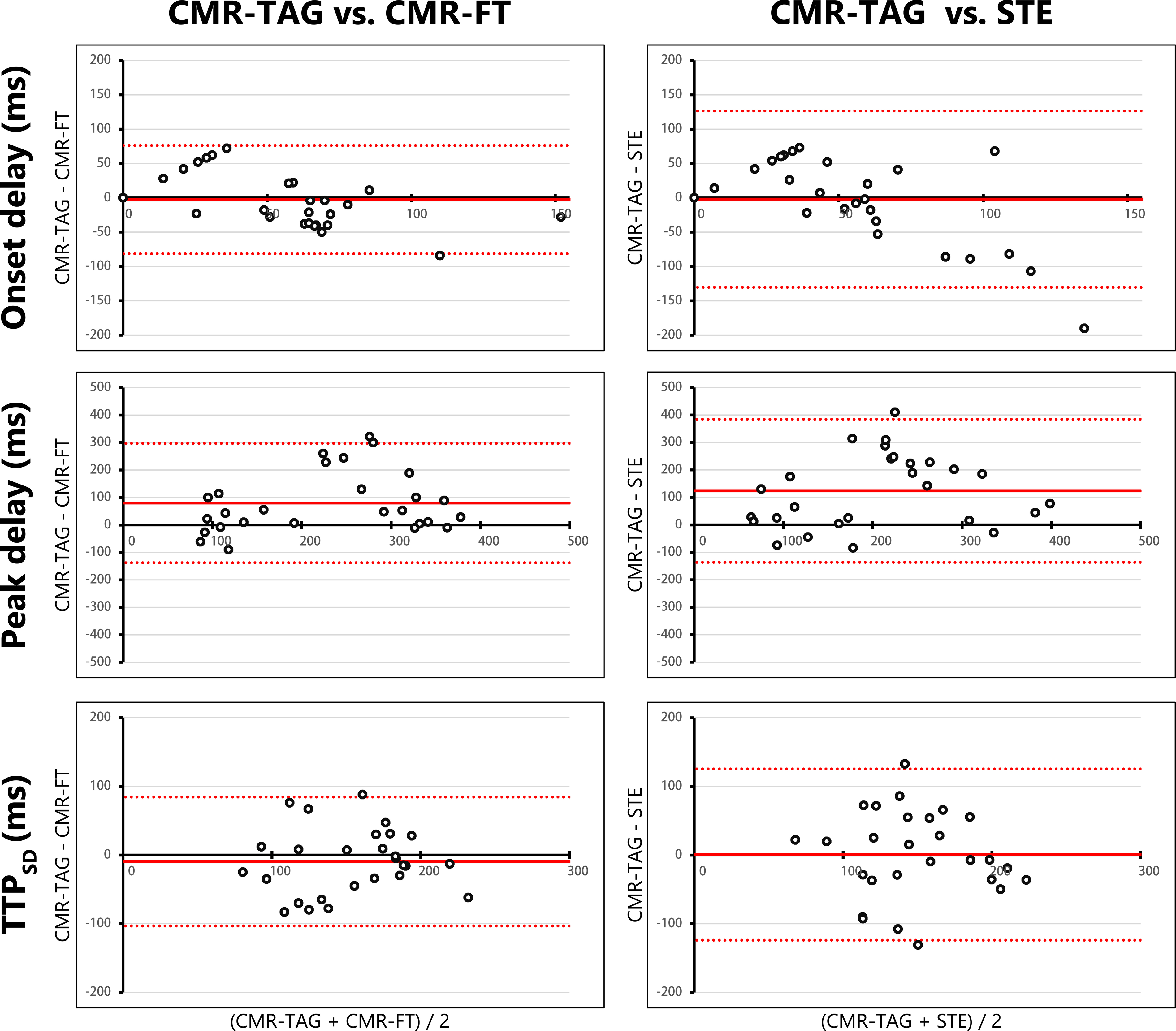

Supplement: Supplementary file 2 — Supplementary material 2 (TIF 1316 KB) [file 10554_2017_1253_MOESM2_ESM.tif]
