# Supplementary material for: Comparison of strain imaging techniques in CRT candidates: CMR tagging, CMR feature tracking and speckle tracking echocardiography
Source: Int J Cardiovasc Imaging. 2017 Oct 17;34(3):443–56. doi: 10.1007/s10554-017-1253-5 (PMC5847211; doi:10.1007/s10554-017-1253-5)
Supplement: Supplementary file 3 — Supplementary material 3 (DOCX 17 KB) [file 10554_2017_1253_MOESM3_ESM.docx]

# Supplemental figures

## Supplemental figure 1. Method for calculation of ISF_LV_

After calculation of the first derivative of strain (panel A), positive and negative strain rate are averaged (red lines, panel B). The areas of positive (dark grey) and negative (light grey) average strain rate during systole are divided to obtain ISF_LV_ (panel C). The end of systole is marked by the black dotted line.

## Supplemental figure 2. Bland-Altman plots of dyssynchrony parameters

Bland-Altman plots for CMR-TAG vs. CMR-FT and CMR-TAG vs. STE of remaining parameters, not displayed in the main manuscript. On the x-axis the mean of two techniques and on the y-axis the difference. The mean difference is displayed as a solid red line, while the limits of agreement are displayed as dotted red lines. Onset delay: delay between onset of shortening of septal and lateral wall. SSI: systolic stretch index. TTP_SD_: standard deviation of time to maximal peak of all segments, CMR: cardiac magnetic resonance imaging, TAG: tagging, FT: feature tracking, STE: speckle tracking echocardiography.

# Supplementary tables

Supplementary table 1. Bland-Altman characteristics of CMR tagging vs. feature tracking and CMR tagging vs. STE.

|  | **CMR-TAG vs. CMR-FT (n=27)** | **CMR-TAG vs. STE (n=27)** |
| --- | --- | --- |
| **Basic strain septum** |  |  |
| 1 - AVC strain septum (%) | 3.5 (-5.2 - 12.2) | 9.2 (-4.0 - 22.5) |
| 2 - Peak strain septum (%) | 1.1 (-4.5 - 6.7) | 6.4 (-3.4 - 16.2) |
| 3 - TTP_max_ septum (ms) | -183 (-656 - 289) | -264 (-754 - 226) |
| 4 - Systolic strain rate septum (%/s) | 8.4 (-16.0 - 32.8) | 21.3 (-11.9 - 54.6) |
| 5 - Diastolic strain rate septum (%/s) | -3.1 (-20.7 - 14.4) | 0.8 (-48.8 - 50.5) |
| **Basic strain lateral wall** |  |  |
| 1 - AVC strain lateral (%) | -0.6 (-7.2 - 5.9) | 1.9 (-9.7 – 13.4) |
| 2 - Peak strain lateral (%) | -1.0 (-6.8 - 4.9) | 2.4 (-9.4 - 14.2) |
| 3 – TTP_max_ lateral (ms) | 19 (-49 - 88) | -50 (-158 - 58) |
| 4 - Systolic strain rate lateral (%/s) | -1.4 (-17.0 - 14.3) | 0.5 (-26.9 - 27.9) |
| 5 - Diastolic strain rate lateral (%/s) | 1.9 (-13.7 - 17.6) | 17.2 (-28.9- 63.2) |
| **Dyssynchrony** |  |  |
| a - Onset-delay (ms) | -3 (-81 - 76) | -2 (-130 - 126) |
| b - Peak-delay (ms) | 80 (-138 - 297) | 124 (-136 - 385) |
| c - TTP_SD_ (ms) | -9 (-103 - 85) | 1 (-124 - 126) |
| **Discoordination septal and lateral wall** |  |  |
| d - SRS_sept_ (%) | 3.4 (-3.1 – 10.0) | 3.7 (-5.3 - 12.7) |
| e - SSI (%) | 3.5 (-3.2 - 10.3) | 3.6 (-4.0 - 11.3) |
| f – ISF_sep-lat_ | 0.14 (-0.245 - 0.53) | 0.19 (-0.16 - 0.53) |
| **Discoordination total LV** |  |  |
| h - ISF_LV_ | 0.11 (-0.22 - 0.45) | 0.10 (-0.31 - 0.50) |
| i - CURE | 0.04 (-0.13 - 0.21) | 0.04 (-0.12 - 0.195) |

Mean difference of the two techniques is given for each comparison (CMR-TAG minus CMR-FT and CMR-TAG minus STE), with the 95% confidence interval within brackets. For abbreviations see table 2.
